# Neuromechanical Cost Functionals Governing Motor Control for Early Screening of Motor Disorders

**DOI:** 10.3389/fbioe.2017.00078

**Published:** 2017-12-13

**Authors:** Midhun P. Unni, Aniruddha Sinha, Kingshuk Chakravarty, Debatri Chatterjee, Abhijit Das

**Affiliations:** ^1^TCS Research & Innovation, Tata Consultancy Services Ltd., Kolkata, India; ^2^Institute of Neurosciences, AMRI Hospitals, Kolkata, India

**Keywords:** optimal control, arm model, cost functionals, early detection, motor disorders

## Abstract

Developing a quantifier of the neural control of motion is extremely useful in characterizing motor disorders and personalizing the model equations using data. We approach this problem using a top-down optimal control methodology, with an aim that the quantity estimated from the collected data is representative of the underlying neural controller. For this purpose, we assume that during the flexion of an arm, human brain optimizes a functional. A functional is defined as a function of a function that returns a scalar. Generally, in forward problems, this functional is chosen to be a function of metabolic energy spent, jerkiness, variance of motion, etc., integrated throughout the trajectory of motion. Current states (angular configuration and velocity) and torque applied can approximately determine the motion of a joint. Therefore, any internal cost functional optimized by the brain has to have its effect in the control of the torque. In this work, we study the flexion of the arm in normals and patient groups and intend to find the cost functionals governing the motion. To achieve this, we parametrize the cost functional governing the motion into the variables *θ_p_* and *ω_p_*, which are then estimated using marker data obtained from the subjects. These parameters are shown to vary significantly for the normal and patient populations. The *θ_p_* values were shown to be significantly higher in the case of patients than in the case of normals and *ω_p_* values showed an exactly opposite trend. We also studied how these cost functionals govern the applied torques in both subject groups and how is it affected while perturbed with sinusoidal frequencies. A time frequency analysis of the resulting solutions revealed a distinguishing pattern for the normals compared with the patient groups.

## 1 Introduction

Primary motor cortex and supplementary motor areas govern the execution and planning of the voluntary motor actions of the skeletal muscles. The projections from primary motor cortex traverse through the corticobulbar and corticospinal tracts and synapse on to the lower motor neurons in the brain stem and spinal cord, respectively. The system of extrapyramidal tracts controls the movement through a non-pyramidal route composing of the nigrostriatal pathway, the basal ganglia, the cerebellum, the vestibular nuclei, and different sensory areas of the cerebral cortex (Hall and John, 2005). Reflexes on the other hand are the result of a hardwired mechanism where interneurons of spinal cord inhibit the stimulation of antagonistic muscles resulting in appropriate movement. Dysfunction of any of these areas can engender a motor deficiency (Kandel et al., 2000). Generally, damage to lower motor neurons produces local effects in the muscles such as weakness, muscle atrophy, and fasciculation. However, upper motor neuron damage can have diffused effects in the body-eliciting spasticity and babinski reflex in adults (Kandel et al., 2000). Quantification of the dynamics governed by the upper and lower motor neurons is a difficult problem.

Computationally, this problem is approached in two ways. First methodology is the use of detailed neural system models with varying complexity (integrate and fire neurons (Brunel and Hakim, 1999), spiking models (Izhikevich, 2003)) to learn the motor control and explain the motor behavior (Grillner et al., 2007; Eliasmith et al., 2012). These methodologies generally use reinforcement learning methods in conjunction, which derives an optimal policy under a set of constraints. Parameter estimation is difficult using these approaches due to (1) their large number and (2) difficulty in defining a tractable cost function. Even in the case where a parameter estimation is possible the models are still not in one to one correspondence with the physiology leading to abstractness in the result.

The second one involves defining an abstract functional, which is optimized to derive the control inputs for the mechanical system (Erdemir et al., 2007). This top-down approach to model the neural controller acts in tandem with the mechanical system that governs the trajectory of motion for a given control input. Therefore, quantifying the motor control involves quantification of two different systems - a mechanical system and the neural controller. These methods, even though abstract, can be solved perfectly to generate results that are optimal.

In this study, we use an approach closer to the second one. The neural controller is known to have characteristics of an optimizer where the cost functional minimized can be jerk, metabolic energy, etc. Therefore, in this study, we use inverse optimal control to find the cost functional that governs the motion. Inverse optimal control refers to a set of techniques that are used to learn the objective functional that governs the system using a data set (Mombaur et al., 2011). These techniques can identify underlying optimality criterion in human motions (Mombaur et al., 2011). In Berret et al. (2011), authors show evidence of composite cost functions governing the arm motion. But this work does not examine normal and patient population and uses numerical techniques that are computationally less efficient. Also, here the arm is modeled using two links rather than three links ignoring the interaction torques that is generated from the third link. The optimal control algorithm used in this model being non-linear also has an issue of non-uniqueness, which makes the applicability of the solution less likely. There is a need to develop a simpler but more efficient technique, so that, it can be used in clinical practice. The method also needs to be reproducible and solution of the optimal control algorithm unique making the results between different patients and at different time frames comparable. A typical reaching task in the horizontal plain neglects gravity. Therefore, the task needs to simulate a real life situation where the arm moves against gravity. This type of modeling although is not a replacement to a more detailed model of neural control, it is useful to obtain personalized neural models. This method overcomes the problems described in detailed models by abstracting away the parameters to be identified. The model-based parameter estimation techniques give insights into the governing motor dynamics and therefore can be used to replace or augment traditional scales used for the quantification. This model can be studied further to understand the behavior of the system against perturbations such as oscillations.

Oscillations are ubiquitous in the brain (Engel and Singer, 2001; Varela et al., 2001), which occur in different scales and at all levels of organization. The organizational levels can range from microscopic (Wang, 2010), mesoscopic (Nunez and Srinivasan, 2006; Cardin et al., 2009) to macroscopic (Llinas et al., 1998; Bollimunta et al., 2011) depending on their source of origin, that is, if oscillations arise from single neurons, a small groups of neurons, or different regions in the brain, respectively.

While intrinsic properties such as conductance govern the oscillations in cellular level (Llinas et al., 1991), the network connection strengths and excitatory-inhibitory nature of the connections control them at network level (Kilpatrick, 2013). A group of neurons engaging in oscillatory activity can be mathematically represented as a lumped system (Haken, 1996). Therefore, in this work, we study how oscillations can affect the motor control.

The primary objective of this study is the development of a methodology to determine a functional governing the motor commands in such a way that it quantifies the neuromotor control abstractly. This method shall give a unique solution for the optimal control problem analytically. This will help in developing an efficient solution that is comparable within and between patient groups at different time frames. The second objective is to validate this model in normal and patient population with mild neuromotor disabilities. (We chose the population with mild neuromotor disabilities as mild neuromotor disability is hard to quantify. The subtle changes in those patients can only be detected by an expert in the field.) The secondary objective is to test the model developed against perturbations and study its effects. We hypothesize that there will be a significant statistical difference between the normal and patient population when the parameters governing the optimal criterion are compared. We also hypothesize that the perturbations of at frequencies around beta frequencies will have higher energy as motor problems manifest around these frequencies.

## 2 Methodology

**Figure 1:**
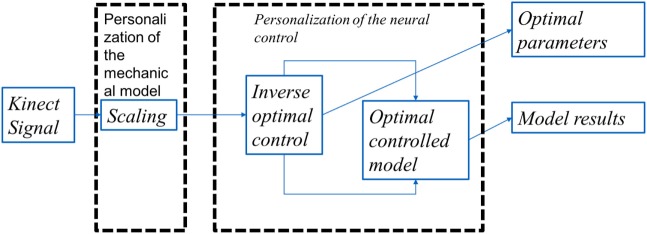
The overall algorithm: the Kinect data after resampling was used as the input to the algorithm. These data were then scaled to correct the model with the joint coordinates of the subject. This was then feed to an inverse optimal control algorithm where the functional **J** is optimized to match the Kinect data. The optimal control in itself involved an optimization of a functional using variational calculus formulation where the search is done in the space of smooth functions matching the boundary conditions.

**Figure 2:**
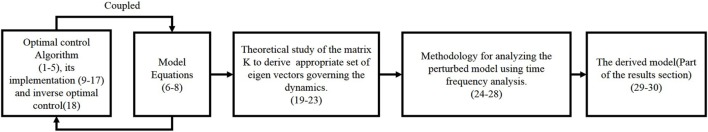
Flowchart shows how the set of equations (1)–(30) are used. The equation numbers are given in brackets. The optimal control and model equations cannot be fully separated to have one causally influencing the other, therefore, it is shown as coupled. The last two equations come from the results section, which depicts the derived functional.

We present the detail methodology of deriving the cost functional governing the arm movement using inverse optimal control. The block diagram for the overall algorithm is given in Figure 1 The transformation matrix is analyzed to understand the principal direction of resistance and a time frequency analysis is done to study the effect of perturbations on the dynamical system. A flowchart is given in Figure 2 with various functional blocks relating with the equations presented in this work.

### 2.1 Optimal Control Formalism

Optimal control formalism relies on the variational formulation (Laub, 1979; Bhattacharyya et al., 2009) of the problem where a functional is minimized under differential constraints. This is particularly useful in situations where the cost has to be described as an integral of a function over a trajectory and the variables of the integrand are governed by system dynamics. A special case of this problem with linear differential constraints is described below.

The problem of minimizing a functional equation (1) with differential constraints equation (2) can be solved in the following way. In equation (2) only **c**(t) is unknown and is the control input, this need to be found in such a way that the cost functional **J** is minimized.

Let the functional to be minimized be

(1) $$\text{J}=\int_{0}^{\infty }\;\left({\text{x}}^{\text{T}}Z\text{x}+{\text{c}}^{\text{T}}U\text{c}\right)\mathrm{dt},$$

where **Z** and **U** are known and let the differential constraint be

(2) $$\frac{d\text{x}}{\mathrm{dt}}=\text{Ax}\left(\text{t}\right)+\text{Bc}\left(\text{t}\right)$$

and **x**(*t*_0_) = **x_0_** then the optimal

(3) c(t)=−Kx(t),

where

(4) $$\text{K}={\text{U}}^{-1}{\text{B}}^{T}\text{S}$$

and **S** is the solution of the following equation

(5) $$-\text{SA}-{\text{A}}^{T}\text{S}+\text{SB}{\text{U}}^{-1}{\text{B}}^{T}\text{S}-\text{Z}=0.$$

This equation is known as algebraic Riccatti equation, which is easy to solve using standard techniques. For each **J**, there is a solution **x** that is optimal.

### 2.2 Mathematical Modeling and Cost Functional

A 3 linked arm model with 3-revolute joints was developed with a sigmoid non-linear constraint function (**k** ∈ **C**^∞^)^1^ imposed on the joints to avoid elbow hyper extension. This constraint is imposed so to restrict the motion in the physiological range. These were rigid links without friction and with shoulder joint as inertial frame of reference.

Control parameter **c**(*t*) are the torques at different joints; gravity is assumed to be acting at center of mass. Kane’s method (Kane et al., 1987) was used to derive the equations of motion (EOM) in the following form:

(6) $$\text{f}+{\text{f}}^{*}=0,$$

where **f** is the generalized active force vector and f* is the generalized inertial force vector. These set of equations were then converted and assembled to the following form and solved using Hindmarsh (1983):

(7) $$\text{M}\frac{d\text{x}}{\mathrm{dt}}=\text{r},$$

where **M** is the mass matrix, **x** is the state vector, and **r** is the forcing vector. Here, the generalized coordinates and speeds were chosen to be the angles and angular velocities, respectively. The system of equation (7) is non-linear. This was linearized at the unstable equilibrium point to convert to the following form. This equation was only used to derive the necessary controller **c**(t). **c**(t) and **c** are used to refer these control inputs interchangeably.

(8) $$\frac{d\text{x}}{\mathrm{dt}}=\text{Ax}+\text{Bc},$$

where **A** ∈ ℝ^6 × 6^ and **B** ∈ ℝ^6 × 3^ represent^2^ the linearized coefficients of the differential equation. The model was scaled for each subject to match the experimentally obtained lengths of the different joints. Equation (9) was then used as the cost functional. First term inside the integral represents cost of change in state **x**, and the second term represents the cost of controls **c**. The final time of the integral is assumed ∞ as the subjects were not given an end time to complete the task.

(9) $$\text{J}=\int_{0}^{\infty }\;\left({\text{x}}^{\text{T}}\text{Z}x+{\text{c}}^{\text{T}}\text{Uc}\right)\mathrm{dt}=\int_{0}^{\infty }{\text{I}}_{v}+{\text{I}}_{c},$$

where

(10) $${\text{I}}_{v}={\text{x}}^{\text{T}}\text{Zx},$$

(11) $${\text{I}}_{v}={\text{c}}^{\text{T}}\text{Uc},$$

(12) $$\text{x}={\left[\theta_{1},\theta_{2},\theta_{3},\omega_{1},\omega_{2},\omega_{3}\right]}^{\text{T}},$$

where *θ*_1_–*θ*_3_ are the joint angles in shoulder, elbow, and wrist, respectively, and *ω*_1_–*ω*_3_ are the angular velocities. **x** is the state variable, **c** is the control torques and **Z** ∈ ℝ^6 × 6^ and **U** ∈ ℝ^3 × 3^ are the weights of independent and controllable variables of the differential equation, respectively. These were constrained to be positive definite. As the function was linearized at the final state x*, and minimization of the cost function **J** also results in minimization of the Euclidean distance between this equilibrium point and the current state. Here, the matrices **Z** and **U** were assumed to be diagonal and arbitrary with the following structure for **Z**.

(13) $$\text{Z}=\left[\begin{array}{cccccc} \theta_{p} & 0 & 0 & 0 & 0 & 0\\ 0 & \theta_{p} & 0 & 0 & 0 & 0\\ 0 & 0 & \theta_{p} & 0 & 0 & 0\\ 0 & 0 & 0 & \omega_{p} & 0 & 0\\ 0 & 0 & 0 & 0 & \omega_{p} & 0\\ 0 & 0 & 0 & 0 & 0 & \omega_{p} \end{array}\right],$$

where *θ_p_* and *ω_p_* represent penalty to angular displacements and velocities, respectively. We assumed **U** = 𝕀^3 × 3^ is the identity matrix making **I***_c_* fixed with respect to which **I***_v_* can be thought to be varied. In the inverse optimal control problem (Figure 1), a requirement for increased penalty to torques applied would translate to an appropriate change in the penalties *θ_p_* and *ω_p_* and therefore determination of the function is given by equation (14)

(14) $${\text{I}}_{v}=\omega_{p}\left(\omega_{1}^{2}+\omega_{2}^{2}+\omega_{3}^{2}\right)+\theta_{p}\left(\theta_{1}^{2}+\theta_{2}^{2}+\theta_{3}^{2}\right).$$

Let Ω and Θ be mean squared values of *ω*_1−3_ and *θ*_1−3_, respectively.

(15) $$\Omega^{2}=\omega_{1}^{2}+\omega_{2}^{2}+\omega_{3}^{2},$$

(16) $$\Theta^{2}=\theta_{1}^{2}+\theta_{2}^{2}+\theta_{3}^{2},$$

(17) $$\Rightarrow {\text{I}}_{v}=\omega_{p}\Omega^{2}+\theta_{p}\Theta^{2}.$$

Making a set of weights constant in relation to another also helps in reducing the search space for the algorithm thus reducing the computational cost considerably. The problem was formulated as an infinite horizon to account the fact that final time was not given to the patients as a criterion while performing the experiments. In summary, the cost functional **J** was solved with differential constraints in equation (8). The inverse optimal control problem of determining the matrix **Z** was done using Nelder and Mead (1965) and Wright (1996). The overall algorithm is depicted in Figure 1.

Mathematically, the optimization problem of finding the personalized J* can be described as in the following way. Each **J** maps to only one trajectory **x**; This makes **x** a function of **J** or f **x**(**J**)

(18) $${\text{J}}^{*}=\underset{\text{J}\in {\text{C}}^{2}}{\text{argmin}}\text{ Er}\left(\text{x}\left(\text{J}\right),\tilde{\text{x}}\right),$$

where **x** is the solution of the optimal control problem for a given **J** and $\tilde{\text{x}}$ is the measured state trajectory. The error function $\text{Er}\left(\text{x}\left(\text{J}\right),\tilde{\text{x}}\right)$ maps the trajectories of the states $\tilde{\text{x}}$ and **x** to the *l*_2_ norm of the difference between the corresponding displacements in the Cartesian y-coordinate of the wrist. The projection of the data in the way described helps to reduce the complexity of the optimization problem significantly in a way that is physically meaningful. The data collection procedure is described in Section 2.5.

### 2.3 Stiffness Ellipsoid or Principal Directions of Resistance in the State Space

The control torques τ depends on the states **x** in the following way:

(19) τ=Kx.

Let

(20) $$\text{K}=\left[\begin{array}{cc} {\text{K}}_{\text{1}} & {\text{K}}_{\text{2}} \end{array}\right],$$

(21) $$\text{x}=\left[\begin{array}{c} {\text{x}}_{\text{1}}\\ {\text{x}}_{\text{2}} \end{array}\right],$$

(22) $$\Rightarrow \tau ={\text{K}}_{\text{1}}{\text{x}}_{\text{1}}+{\text{K}}_{\text{2}}{\text{x}}_{\text{2}},$$

where **K** ∈ ℝ^3 × 6^ is the transformation matrix, ***x***_**1**_ and ***x***_**1**_ are the angles and angular velocities, respectively. For constants ***K***_**1**_ and ***K***_**2**_ and ***x***_**1**_, the value of ***x***_**2**_ determines the value of applied torque. We define

(23) $$\tau^{*}=\underset{{\text{x}}_{\text{2}}\in {\mathbb{R}}^{1\times 3}}{\text{max}}\;∥\text{Kx}∥,$$

where ***x***_**2**_ is the vector of angular velocities. The resistance torque applied by the neuromuscular system depends on two characteristics of the angular velocity vector **x**. First one, the magnitude of the angular velocity vector, which will directly increase the torque applied. The second one is the direction of the angular velocity vector. We denote this direction by ${\text{x}}_{\text{2}}^{*}$ and this is the principal eigenvector of the matrix ***K***_**2**_. If angular velocities are perturbed in this direction the resistance offered by the neuromechanical system peaks. This torque vector acts solely against the angular velocities resisting a higher velocity of motion. The largest component of this vector is indicative of the joint velocity that is resisted the most and the difference these eigen values among subjects represent intra-subject variability in resistance to motion.

### 2.4 Frequency Response and Time Frequency Analysis

To understand the response of the dynamical system behavior against perturbations, we studied the response of patient and normal systems to spectral perturbations. The new set of equations governing the perturbed system is same as that is described previously except equation (3), which is replaced by equation (24)

(24) $$\text{c}\left(t\right)=-\text{K}{\text{x}}_{n}\left(t\right),$$

(25) $${\text{x}}_{n}=\text{x}+\lambda \cos(\gamma t)),$$

(26) $$\lambda =\kappa *I_{6\times 6},$$

where **x***_n_* is the “perturbed” state signal, which represents different oscillations that arise in the brain and 𝕀_6 × 6_ is an identity matrix. The resulting solution of the optimal dynamical system for different *γ* values was computed, and a time frequency analysis of the solutions was performed using equation (27). *κ* (*κ* = 0.2) was chosen to be of a value that produces physiological relevant ranges for the results and easy to manage numerically.

(27) $$W\left(t,f\right)=\int \text{x}\left(t+\tau ∕2\right){\text{x}}^{\prime }\left(t-\tau ∕2\right)e^{-j2\pi \tau f}d\tau .$$

The function *W* (*t*, *f*) corresponding to each solution **x** is analyzed the following way by using equation (28) to understand the time frequency pattern against different frequency of inputs. Here, τ is an arbitrary delay variable.

(28) $$E_{t,f}=\int_{0}^{f}\int_{0}^{t}W\left(t,f\right).$$

The *E_t_*_,_*_f_* was computed for a time window of 60 s for all frequencies estimated. This limit was chosen to understand the effect of the frequencies during the motion rather than at the trailing end of the bell curve of velocities.

### 2.5 Data Collection Using Kinect and Signal Processing

The range of motion for left shoulder flexion is collected using Microsoft Kinect XBox One.^3^ A total of 13 normal subjects and 19 patients with neuromuscular disorders were selected for the study. Each session was repeated four times for all normal subjects resulting in total of 52 samples whereas a single trial was used for patients generating total 19 samples.

The clearance on ethical issues corresponding to the patient data collection has been obtained from the Institute Ethics Committee (IEC) in Advanced Medical Research Institute (AMRI) Hospitals. Informed consents are also taken from the subjects in written form. The data are annonymized by representatives of AMRI in a community setup and provided to TCS Research Lab for purpose of the analysis presented in this paper. The clearance on ethical issues corresponding to the handling and analysis of the data collection has been obtained from relevant body in TCS. The patients were of the age group range 56–74 with a mean of 65.6 and a SD of 4.62. The normal subjects were of the age group 22–45 with an SD of 10.3 and mean of 30.1. For the healthy group male, the average height was 172 (163–183) cm, and for the females, it was 158 (150–161) cm. For male patients, the average height was 171 (164–180) cm, and for the female patients, it was 160 (156–170) cm. The average weight of the healthy male group was 78.25 (70–84) kg, and for the female group, it was 63.5 (56–68) kg. The average weight of the patient male group was 74.83 (62–90) kg and that of the female patients was 67.2 (60–74) kg. The patient group had mild neuromotor abnormalities due to diabetes and old age.

These data were then resampled from 30 to 60 fps for the analysis using built-in python Fourier method. This resampling was done to make the numerically generated trajectory vector, and the data collected have the same dimension. The lengths of different bone segments were then computed for each subject using the 3D coordinates of the skeleton data of the initial 5 s of static window before the start of the exercise. This was used for scaling, and the scaled model was given as input to the inverse optimal control algorithm to optimize the cost functional.

## 3 Results

**Figure 3:**
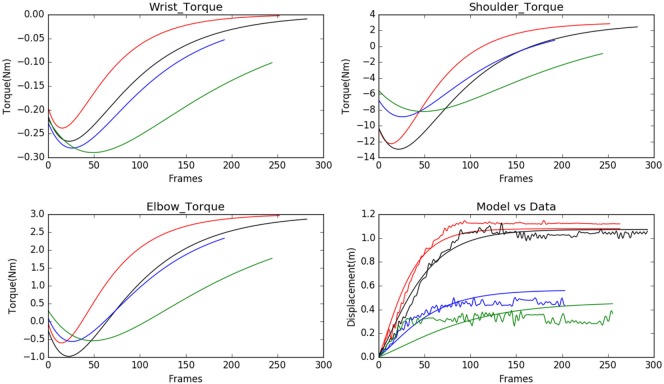
Model predicted torques for wrist, shoulder, and elbow joints for 4 normal subject samples are shown (each of the subjects is assigned a single color in the graph). The model is personalized to match the experimental observations of displacement. The range of motion is very well matched by the model used.

**Figure 4:**
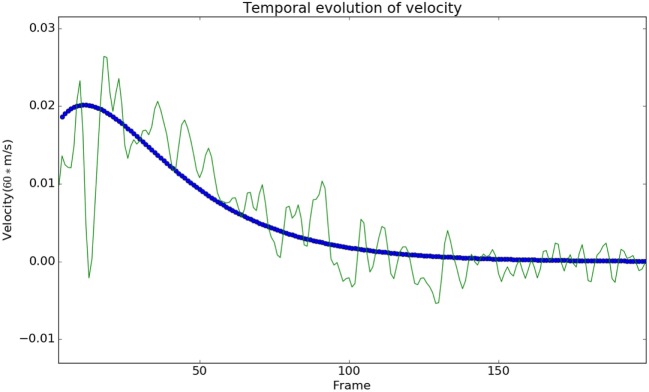
The modeled velocity profiles match qualitatively with the Kinect data. The deviation of the Kinect data from the model is due to the fact that the process of finding the instantaneous velocity involves taking numerical derivatives, which amplify the high-frequency noise. Modeled velocity is in blue, and the data are shown in green.

We observed that the displacements obtained from the model matched with the experimental results from the Kinect with negligible error, see Figure 3. In Figure 3, each subject is represented using a single color. The subfigure of Figure 3 titled Model vs Data shows how the modeled displacement is matched to the measured displacements (noisy due to the measurement noise) using the optimization procedure given in Subsection 2.2 using equation (18). The computed velocity is also shown to match the measured values as shown in Figure 4. This verifies the ability of the model to approximately reproduce the kinematics of the subjects.

**Figure 5:**
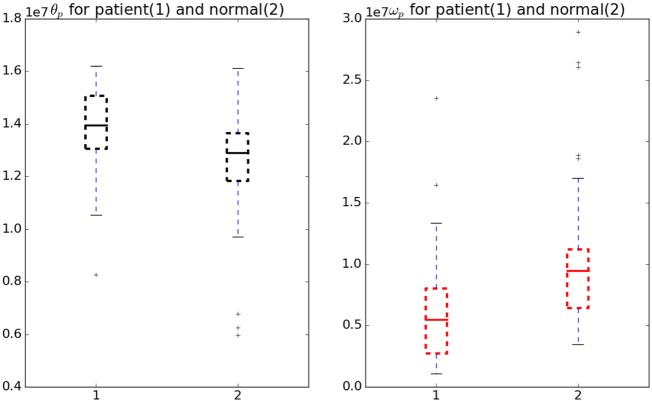
Variation in *θ_p_* and *ω_p_* for normal and patient categories. The *θ_p_* (black) is significantly higher in patients than in normals whereas *ω_p_* (red) is significantly lower in patients than in normals.

Figure 5 shows the comparison of the *θ_p_* and *ω_p_* in normal and patient categories. To analyze the significance, a Welch’s t-test (Welch, 1947) was performed without assuming equal variance or equal number of samples (Ruxton, 2006). We found these cost-function parameters vary significantly between normal subjects and patients (see Figure 5) with a test statistic value of −2.28, p-value of 0.027 and test statistic value of 2.3, p-value of 0.023 for *ω_p_* and *θ_p_*, respectively.

The test used did not assume the equal variance, which if assumed the p-value will be lower. Therefore, these parameters can be considered as a good measure to understand the underlying optimality criterion for the motor control mechanism of a patient. A lower *ω_p_* was observed in patients indicating jerky and fast movements. On the other hand, a higher *θ_p_* change was observed in patients indicating a lower ability to hold the hand at intermediate position. There are two objectives for this study, first one being the detection of optimal functions governing the neuromotor control and the second one finding the response of the model against perturbations.

### 3.1 Optimal Functions—Patient and Normal

Here, we detail the optimal functions of the normals and patient groups. Using the optimal control equations described in the methods sections, we estimated the functions **I***_v_* for the normals (**I***_vn_*) and the patients (**I***_vp_*), which take the forms described in equations (29) and (30).

(29) $${\text{I}}_{\mathrm{vn}}=9,315,830\Omega^{2}+12,926,292\Theta^{2},$$

(30) $${\text{I}}_{\mathrm{vp}}=5,245,197\Omega^{2}+13,950,617\Theta^{2}.$$

**Figure 6:**
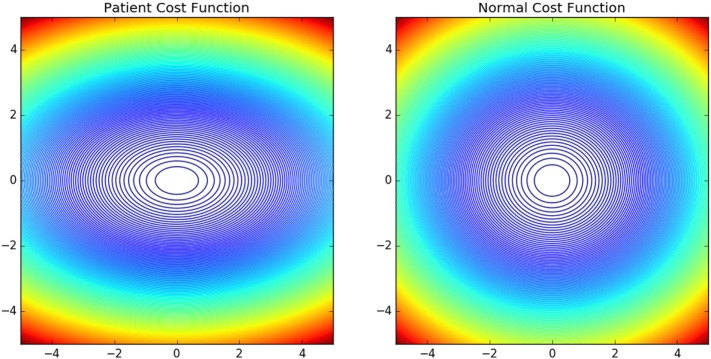
Shows the contour plot of **I***_v_*(Θ, Ω) of normals and patients with abscissa show change in Ω and ordinate change in Θ. The normal **I***_v_* is more circular compared with the patient **I***_v_*. This owes to the fact that the motion is more smoother and of lower velocity in normals compared with the patient group.

**Figure 7:**
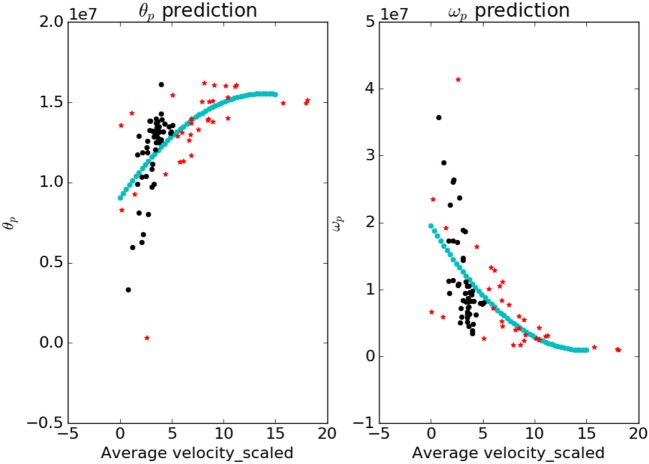
The variation of parameters *θ_p_* and *ω_p_* with respect to the average velocity is shown. The blue curve is a fit that predicts the value of parameters for a given value of average velocity. The values of *θ_p_* are higher for the patients and lower for the normals; *ω_p_* shows an opposite trend.

**Figure 8:**
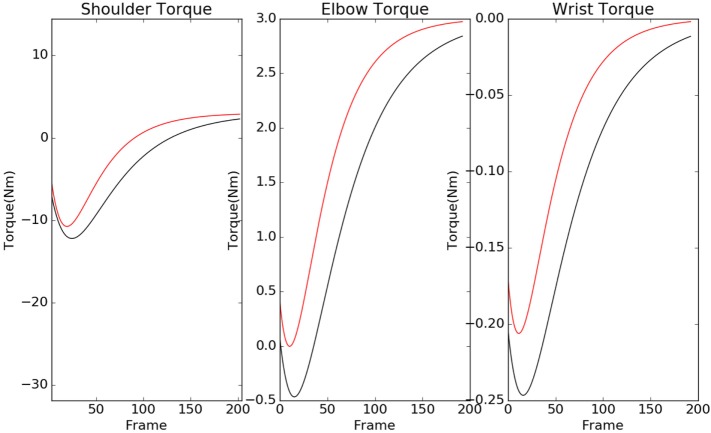
Torques of the normal (black) and patient (red) are shown here for shoulder, elbow, and wrist. The patients tend to use a higher torque than normals, and shoulder torque has a higher range in compared with elbow and wrist.

The contour plots of **I***_vp_* and **I***_vn_* with respect to different values of Θ and Ω are shown in Figure 6. The contours in the case of normals are more circular than that of the patients. This is due to the fact that ratio of *ω_p_* to *θ_p_* is lower in the case of patients than in the case of normals. This increases the tendency to move faster in the case of the patients leading to a more jerkier motion and inability of the patients to slowdown in an intermediate position. Figure 7 shows how in different subject groups the *θ_p_* and *ω_p_* varies in detail. Here, each point represents a subject. The value of *θ_p_* is higher in the case of patients (shown in red) and lower in the case of normals (shown in black). Figure 7 also shows the pattern of *θ_p_* and *ω_p_* with respect to the average velocity of motion. An increased *θ_p_* and a lowered *ω_p_* show similar effects on the average velocity. The optimal torques for both patient and normal cases are shown in Figure 8. As expected from the cost functions, the normals use lesser torques than that of the patients for reaching the same targets. The shoulder experiences maximum range of torque inputs compared with the elbow and wrist. This higher range of torques in the shoulder helps it resist the higher moments against which it moves.

### 3.2 Response to Oscillations and the Eigen Values

**Figure 9:**
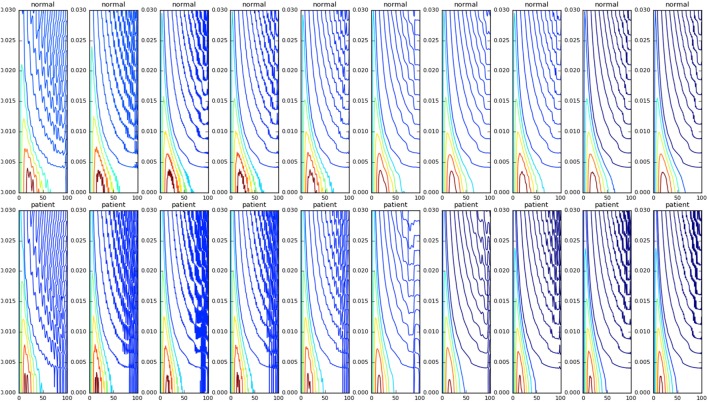
The WVD of the velocity waveforms is shown. The patient group tends have a higher energy at the start of the motion compared with the normals.

**Figure 10:**
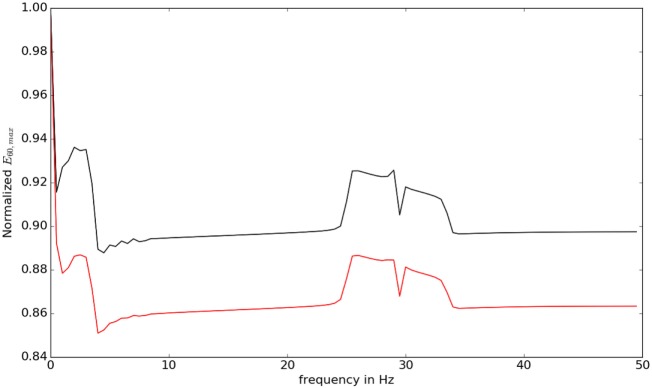
The value of $\int_{\text{0}}^{\text{30}}\int_{\text{0}}^{\text{60}}W\left(t,f\right)$ for different frequencies is shown. At low frequencies and close to beta oscillation frequencies, the response of the system is observed to be higher.

We then studied the response of the system. The time frequency analysis performed using equation (27) showed and increased energy in earlier in time frame than later as shown in Figure 9. The energy levels, computed using equation (28), are higher at lower frequencies and around the frequencies that correspond to the beta oscillations. To illustrate this, we have computed *E_t_*_,_*_f_* for *t* = 60 and *f*  = *max* (as there is a bound to the maximum frequency content that can be estimated) was computed, normalized by dividing with the maximum value of the response and plotted in Figure 10.

**Figure 11:**
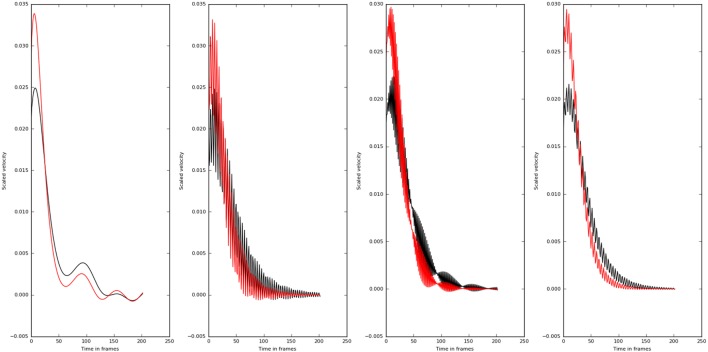
Velocity profiles of motion corresponding to different frequency of inputs (0.5, 15.5, 30.5, and 45.5 Hz left to right) are shown. The higher frequencies do not affect the velocity profiles as much as the lower frequency values. Also there is a qualitative change in the velocity profiles at 30.5 Hz. The normal subjects are shown in black, and the patients are shown in red.

Even though, all the principle directions remained the same for patient and normal groups, normals had lower eigen values compared with the patients. This shows why normal population apply lesser torques compared with the patient. The eigen directions ${\text{x}}_{\text{2}}^{*}$ remained the same for the normals and patients. The wrist velocities corresponding to the input oscillation at frequencies 0.5–45.5 Hz are shown in Figure 11.

## 4 Discussion and Future Research

The objective of this work was to develop a methodology to quantify and understand the neuromechanical pathologies and validate it using the methodology on two subject groups one normal and another with mild neuromotor abnormalities. We found that the inverse optimal control methodology can be used to personalize model of the arm dynamics. Thus, the cost functional **J** parameterized by *θ_p_* and *ω_p_* can be used to understand subject specific variations. We also have showed how the eigen vectors of the matrix ***K***_**2**_ controls the torque applied. The different eigen values controlling the resistant torques are indicative of the higher resistance offered by the normals against a drastic change in velocity. This protects the population against natural wear and tear. In Figure 6, for the patient case moving through the ordinate will reach the maximum values (shown in red) compared with moving through the abscissa. We also note that in the case of normals as the cost function is more steeper ease of reaching the minima is more faster. It can be speculated that in a noisy case, this will govern the ease with which motor control decisions are taken making the control in case of the patients more difficult.

The lower frequencies and the frequencies around 30 Hz show an elevated response eliciting the differential response of the controller against different frequencies. This also indicates why some oscillations in the brain can result in motor symptoms.

In future, we intend to collect data from patients with neuromuscular disorders and estimate the parameters as the disease progresses. We argue that reversal of the parameters from diseased states to the normal (see Figure 5) is a necessary condition to validate treatment efficacy (from elliptical to normal). The cost functional can be further generalized, and a detailed set of parameters can be estimated to better understand the physiology. Careful considerations are needed from optimization front while generalizing the cost functional as this will increase the dimensionality of the problem.

Even though we have used a simple planar 3-link arm that is not a major limitation of the study as the task was constrained to a planar motion. But constraining the task to a planar motion may not be able to reveal all aspects of proprioception and motor control but this helps in making the task easily reproducible in a clinical setting. Another limitation is the linearization that was carried out to make the solution unique for the optimal control problem. Linearizing at different points, solving the optimal control problem and comparing the results will resolve this limitation. Extending the model to a realistic motion involves adding more dimensionalities to shoulder, elbow, and wrist joints and constraining the motion realistically. Although, this not necessary to simulate a planar motion could be used for other tasks such as pick and place. But this will render the problem of optimization computationally expensive. But simulating and doing the optimal control on such a task may provide very valuable information to the clinician such as how different muscles are affected by the neuromotor disorders. This will help in designing a task to strengthen the appropriate muscles. The shoulder flexion task involves movement of the arm against gravity. Most of the practical real life scenarios such as combing hair involve movement against gravity. *θ_p_* and *ω_p_* are abstract quantities. Supplementary motor area and other areas of the brain involved in planning of the motion directly affect the magnitude of these quantities. Velocity-dependent conditions such as hyper reflexia and spasticity can directly affect the eigen values of the matrix ***K**_2_* and thus resistance against velocities.

More precisely, an ischemic stroke in the middle cerebral artery (MCA) affecting the motor cortex or sensory cortex will have an impact on the parameters mentioned. The parameters can also be affected by stroke in subcortical regions which in turn result in motor deficits. In addition, the parameters could be influenced by aging-related changes such as changes in spindle innervation, increased co-contraction of agonist and antagonist muscles, and decreased reaction times due to decreased motor conduction. Precisely, mapping different disease conditions to parameters is a future work.

Noise in smaller levels will not affect the computation of these parameters as the differential equation can only give smooth solutions, the method will act as a filter. But in cases where the noise is very high and skewed will contribute to the study results. A careful study may be conducted later to understand the effects of noise in the analysis.

## Ethics Statement

The clearance on ethical issues corresponding to the patient data collection has been obtained from the Institute Ethics Committee (IEC) in Advanced Medical Research Institute (AMRI) Hospitals. Informed consents are also taken from the subjects. The data are annonymized by representatives of AMRI in a community setup and provided to TCS Research Lab for purpose of the analysis presented in this article. The clearance on ethical issues corresponding to the handling and analysis of the data collection has been obtained from relevant body in TCS.

## Author Contributions

MU conceived the model, developed it, analyzed the data, and prepared the manuscript. AS conceived the model and prepared the manuscript. KC, DC, and AD designed the experiment and collected the data.

## Conflict of Interest Statement

MU, AS, KC, and DC are employed as researchers by TCS Research and Innovation, Tata Consultancy Services Ltd. AD is the Director of Neurorehabilitation Senior Consultant Neurologist, AMRI Institute of Neurosciences Kolkata. He received honorarium as a research advisor from TCS Research and Innovation, Tata Consultancy Services Ltd. All the authors declare no other competing interest.

## Footnotes

^1^**C***^n^* is the set of all *n* times differentiable functions.

^2^ℝ*^m×n^* is the set of all *m* × *n* matrices.

^3^http://www.xbox.com/en-IN/xbox-one/accessories/kinect.
